# Managing the Open Abdomen in Damage Control Surgery: Should Skin-Only Closure be Abandoned?

**DOI:** 10.7759/cureus.15489

**Published:** 2021-06-07

**Authors:** David M Milne, Amrit Rambhajan, Jason Ramsingh, Shamir O Cawich, Vijay Naraynsingh

**Affiliations:** 1 General Surgery, General Hospital Port of Spain, Port of Spain, TTO; 2 General Surgery, Royal Victoria Infirmary, Newcastle upon Tyne, GBR; 3 Surgery, The University of the West Indies, St. Augustine, TTO; 4 Clinical Surgical Sciences, The University of the West Indies, St. Augustine, TTO; 5 Surgery, Medical Associates Hospital, St. Joseph, TTO

**Keywords:** damage control laparotomy, damage control surgery, temporary abdominal closure, laparostomy, trauma surgery

## Abstract

During damage control laparotomy, surgery is abbreviated to allow for the correction of physiologic disturbances, with a plan to return to the operating theatre for definitive surgical repair. Re-entry into the abdomen is facilitated by temporary abdominal closure (TAC). Skin-only closure is one of the many techniques described for TAC Numerous sources advise against the use of this technique because of the risk of complications. This case report describes the use of skin-only closure during a damage control laparotomy. We reviewed the literature surrounding the various options for TAC to elucidate the potential role of skin-only closure after damage control laparotomy.

## Introduction

In 1983, Stone et al. described his experience with 17 patients who developed significant bleeding diatheses during laparotomy [1]. Instead of completing the laparotomy, he made attempts to arrest haemorrhage, then packed the abdomen with laparotomy pads and closed the abdominal wall under tension. After correction of coagulopathy, patients were returned to the operating room to complete definitive surgery. His success with an abbreviated laparotomy, with a plan to re-operate, sparked a revolution in trauma surgery.

Following on from the experience of Stone et al., Rotondo et al. coined the term damage control in 1992, when he treated patients with exsanguinating penetrating trauma to the abdomen with initial haemorrhage and contamination control, abdominal packing, and temporary abdominal closure (TAC) [2]. This was followed by resuscitation in the intensive care unit (ICU) and subsequent definitive re-exploration. The period of resuscitation allowed for the correction of coagulopathy, hypothermia, and acidosis. Using this damage control approach, resulted in decreased mortality in patients with two or more visceral injuries and major vascular injuries [2].

Damage control approaches have now been widely adopted as a useful treatment strategy in managing appropriately selected, multiply injured patients. However, damage control surgery (DCS) is associated with increased morbidity compared to single-stage procedures [3]. This has led to some trauma centres taking measures to reduce their rate of DCS [4].

Major contributors to morbidity after DCS include abdominal compartment syndrome (ACS) and the consequences of delayed fascial closure. Both these complications are influenced by the technique utilised to achieve TAC [5].

Many different techniques have been described for TAC. They include skin-only closure with suture or towel clips, Bogotá bags, mesh closure, Wittmann’s patch, and negative pressure therapy (NPT). There have been numerous calls in the literature to abandon skin-only closure as a TAC technique to manage the open abdomen (OA) because of a high risk of complications [6-11]. The heterogeneity of the literature hinders evidenced-based decisions on which type of TAC to employ. Consensus guidelines have recommended NPT as the technique of choice [5,7,12]. Unfortunately, in low-resource environments, vacuum-assisted closure may be unavailable, and surgeons must adjust their practice.

Here, we report a case of DCS for penetrating trauma that was managed with towel clips to close the skin as a means of TAC. The discussion will explore the literature surrounding the various techniques for TAC to determine whether calls for the abandonment of skin-only closure are justified.

## Case presentation

**Figure 1:**
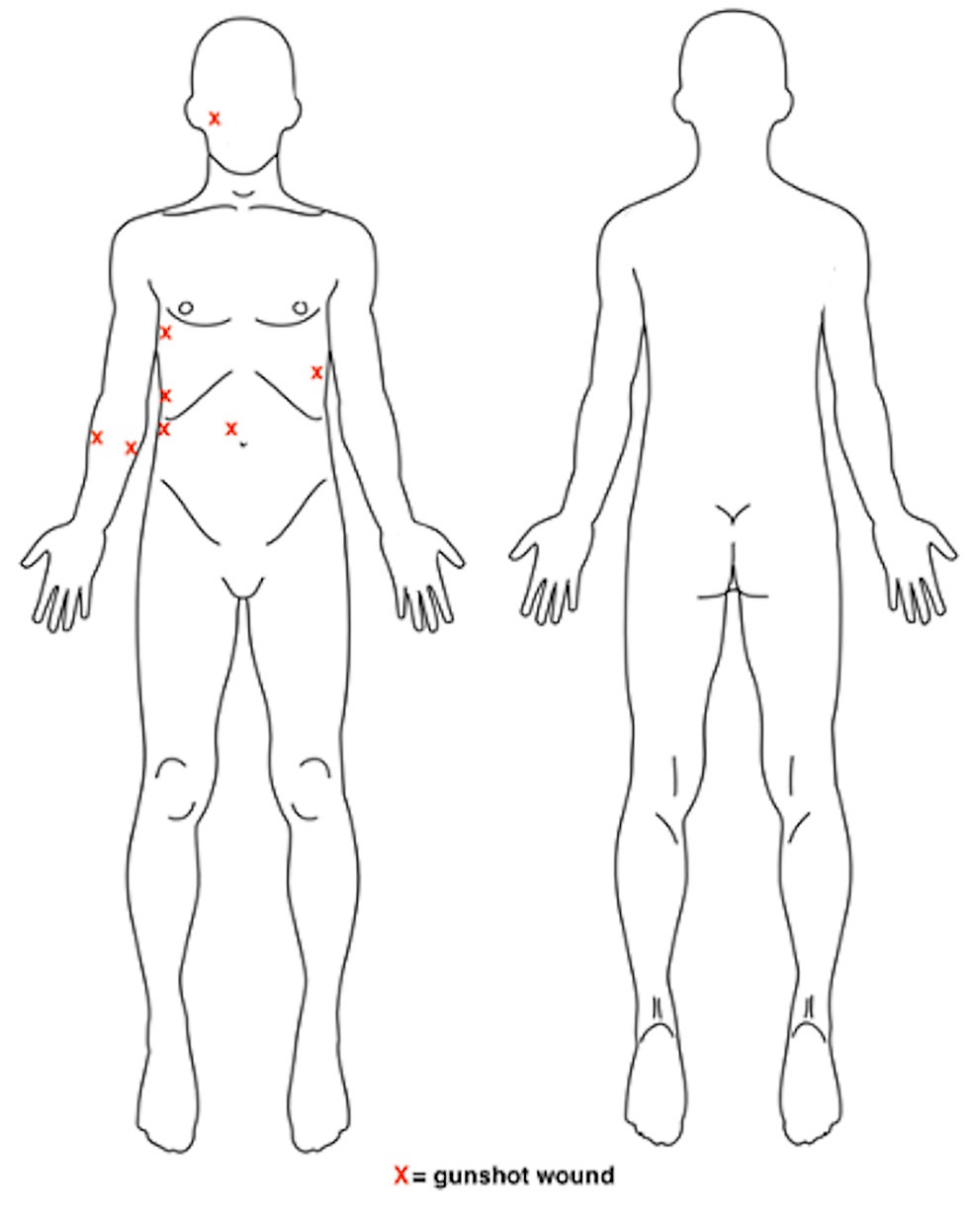
Gunshot wounds seen on examination (marked by red X)

A 32-year-old male presented in extremis with multiple gunshot wounds to the abdomen, chest, right upper limb and face (Figure 1). On admission, his pulse was 142 beats per minute, blood pressure was 61/40, and respiratory rate was 36 breaths per minute. His Glasgow coma scale score was 11/15, which deteriorated from 15/15 during transport.

Examination of the mouth and oral cavity found no abnormalities. Decreased air entry was noted bilaterally with associated dullness to percussion. Bilateral thoracostomy tubes were placed, draining 800 ml of blood on the right and 200 ml of blood on the left. His abdomen was distended with generalised tenderness, guarding, and rebound. Pupils were symmetrical and reactive to light.

High flow oxygen was commenced via a non-rebreather mask. Two 16-gauge intravenous catheters were placed, a bolus of Ringer's lactate commenced, and uncross-matched blood requested. Routine blood investigations and arterial blood gas sampling were done, which revealed a pH of 7.1. A bolus of 1 g of tranexamic acid was given over 10 minutes, followed by a 1 g infusion set to be administered over eight hours.

Considering his hypotension, acidosis, and the possibility of intracranial haemorrhage from the gunshot wound to the face, he was taken to the operating theatre for laparotomy with the expectation that a damage control approach would be required. A midline incision was performed from xiphisternum to pubic symphysis. Two litres of blood were evacuated from the abdominal cavity. An American Association for the Surgery of Trauma (AAST) grade IV liver laceration was noted affecting Couinaud segments III, IV, V, VII, and VIII. The liver was packed anteriorly and posteriorly with eight laparotomy sponges which achieved haemostasis.

**Figure 2:**
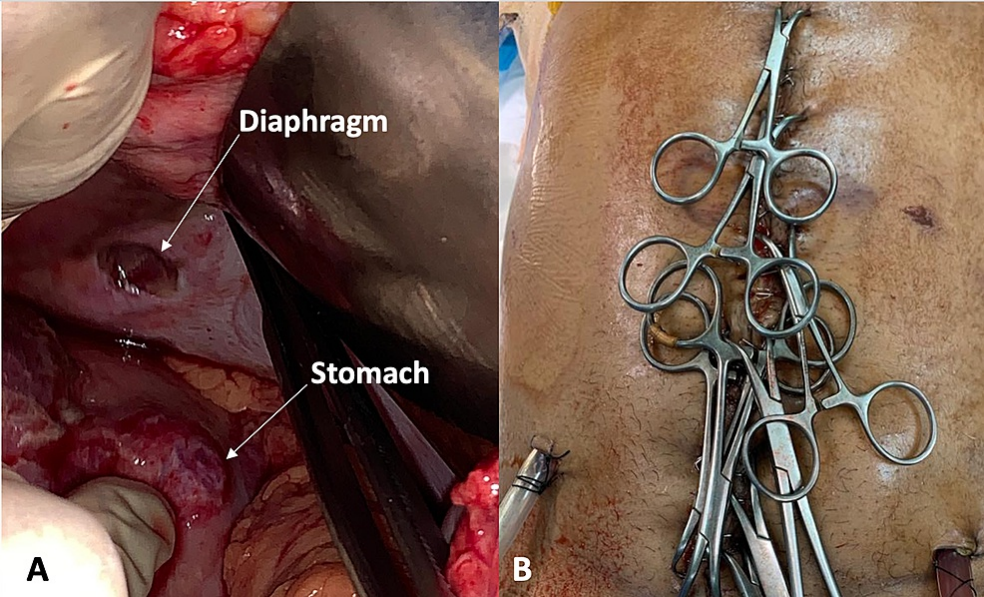
Intraoperative pictures from damage control laparotomy (A) Lacerations to diaphragm and stomach; (B) temporary abdominal closure with towel clips

Two AAST grade II lacerations were noted on the lesser curvature and body of the stomach, approximately 1.5 cm in length (Figure 2a). These lacerations were closed with interrupted 0 silk suture. Two AAST grade IV and one AAST grade II lacerations of the jejunum were ligated using a 5 mm polyester fibre ligature. One AAST grade II laceration to the left anterior costal part of the diaphragm was identified, and repair was deferred (Figure 2a).

During the operation, two units of uncrossed matched blood were transfused, and triple inotropic support initiated. His systolic blood pressure rose to 90 mmHg, allowing for examination of the peripheral pulses, which were intact. No further bleeding was noted from the thoracostomy tubes, and there was no bulging of the diaphragm. Haemostasis was confirmed before TAC was undertaken using towel clips (Figure 2b). At the end of the operation, his temperature was 35 °C.

**Figure 3:**
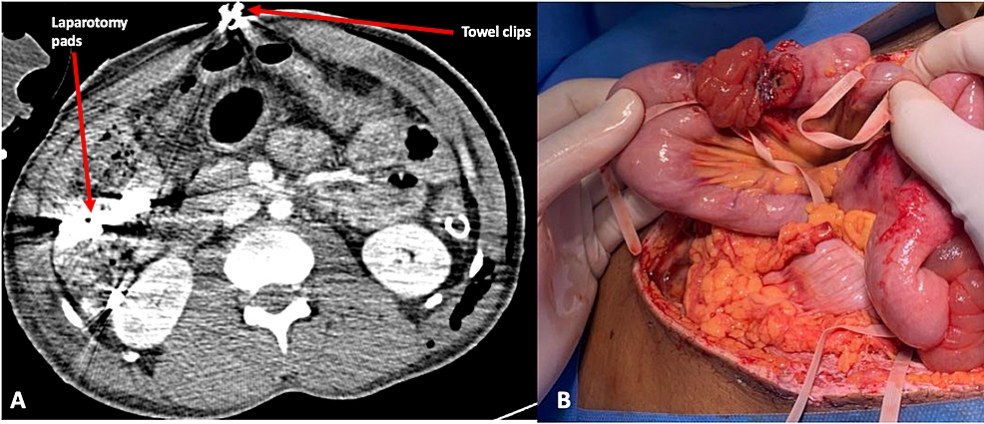
CT findings and intraoperative picture from second-look laparotomy (A) Artefacts seen on CT from towel clips and laparotomy pads; (B) intact bowel ligatures at time of the second laparotomy

Resuscitation was continued in the ICU with packed red cells and fresh frozen plasma. Computed tomography (CT) of the brain, chest, abdomen, and pelvis was done. A right intraorbital bullet was noted with no evidence of intracranial haemorrhage. The heart and imaged great vessels were normal. Intra-abdominal packs were noted. There was no evidence of contrast extravasation in the abdomen to suggest continued haemorrhage. Interpretation of CT images was hindered by artefacts produced by the towel clips used in TAC and laparotomy pads used in visceral packing (Figure 3a).

Transvesical intrabdominal pressure monitoring revealed normal intrabdominal pressures throughout his ICU stay. Over the next 48 hours, his pH, body temperature, prothrombin time, partial thromboplastin time, and haemoglobin were returned to the normal range with appropriate resuscitation and blood product administration. His inotropic support was successfully weaned, and he was subsequently taken back to the operative theatre for definitive repair of his injuries.

Intrabdominal packs were removed, revealing no evidence of ongoing haemorrhage. The polyester fibre ligatures were intact with no evidence of enteric soiling of the peritoneum (Figure 3b). These ligatures were removed, and the edematous bowel debrided. The bowel was repaired using a 2.0 polyglactin suture in two layers. The diaphragmatic injury was repaired using a 0 nylon suture in an interrupted fashion. A drain was left in Morison’s pouch to treat the expected biliary leak. Primary fascial closure was done using 0 looped nylon sutures, and the skin was loosely approximated with 2.0 polypropylene sutures.

Bile-stained fluid was noted from the abdominal drain in the post-operative period. This resolved after one week. Return of bowel function occurred on post-operative day 4, with the patient tolerating oral intake. The rest of his post-operative course was otherwise uneventful. There was no clinical evidence of incisional hernia 10 months postoperatively.

## Discussion

OA is a term used to describe the process of deliberately leaving the fascial edges of a laparotomy wound open to form a laparostomy. OA may be used to shorten the operative time, facilitate abdominal re-exploration without additional fascial trauma, and prevent or treat intraabdominal hypertension. TAC is how abdominal organs are protected while the fascia remains open [12]. In 1940, Ogilvie published the first report on OA and TAC, where he used canvas to bridge facial defects in wartime laparotomy wounds that had too much tension to close primarily [13].

The indications for OA can be broadly categorised as physiologic, anatomic, and logistical. Physiologic indications occur when haemodynamic instability secondary to severe physiologic disturbances mandates a damage control laparotomy. Anatomic indications occur when the closure of the abdomen is precluded by soft tissue loss (from trauma or sepsis) or a high risk of ACS. Logistical indications pertain to patients in whom multiple abdominal surgical interventions are necessary, so the abdomen is left open to preserve the fascia [14].

OA is most commonly utilised in trauma when DCS is performed, as demonstrated in the index case. It may also be necessary in trauma cases when there is significant tissue loss from the abdominal wall precluding closure, after mesenteric vascular injury to facilitate a second-look laparotomy, to avoid ACS in high-risk cases (significant oedema at the end of laparotomy, visceral packing, elevated bladder pressures during fascial closure), to treat secondary ACS, and to facilitate removal of visceral packs [15].

OA is also used in non-trauma surgery. Indications include septic shock with haemodynamic instability mandating an abbreviated laparotomy, necrotising infections involving the abdominal wall, haemodynamic instability during the treatment of ruptured abdominal aortic aneurysms and other vascular catastrophes, planned second-look laparotomy after surgery for acute mesenteric ischemia and in the treatment of ACS [16,17].

OA is a practical approach to managing certain patients, but it comes with a significant risk of morbidity. Large fluid and heat losses occur via laparostomy. Patients can lose multiple litres per day, which is a challenge to both fluid balance and nursing care. The fluid lost is protein-rich, with on average 2 g of protein lost per litre of abdominal effluent [18]. The frequent manipulation of the bowel predisposes it to trauma, placing the patient at risk of developing enterocutaneous fistulae, which form in as many as 20% of cases [19,20]. OA leads to fascial retraction due to the lateral pull of the abdominal musculature, which can interfere with primary fascial closure. After OA, only 34-74% of patients can achieve primary fascial closure, with the remainder condemned to an incisional hernia [21]. Given the high risk of morbidity associated with OA, surgeons should identify ways to modify this risk.

The method of TAC directly influences the complication rate observed in the OA [12]. Therefore, the selection of the TAC technique is a critical decision that can influence patient outcomes. The ideal method of TAC has several features which one should consider when making a choice. It should serve as an effective barrier, preventing evisceration and external contamination, allowing for patient transport. It should allow for the egress of peritoneal fluids in a controlled manner, preventing excessive losses while allowing quantification of losses and easy nursing care. It should limit abdominal wall retraction and prevent loss of domain to facilitate primary fascial closure while still allowing for expansion to prevent ACS. It should prevent damage to the bowel to minimise the risk of fistula formation and reduce adhesions. It should be easily and rapidly performed. It should allow for easy entry into the abdomen for re-exploration. It should be readily available and inexpensive [6,9,22,23].

Unfortunately, no single TAC method can meet all these desired features, leaving the surgeon the task of weighing the merits of each technique and applying the one that best fits their institution’s and patient’s circumstances. The existing literature on OA is heterogeneous, and it is difficult to formulate definitive guidance. This is highlighted in The World Society for Emergency Surgery’s guidelines on the OA [12]. They state that the guidelines present methods for optimal management of patients but should not be considered a standard of practice, and readers should not exclude other approaches [12]. With this in mind, we will review the merits of the various options available for TAC.

**Figure 4:**
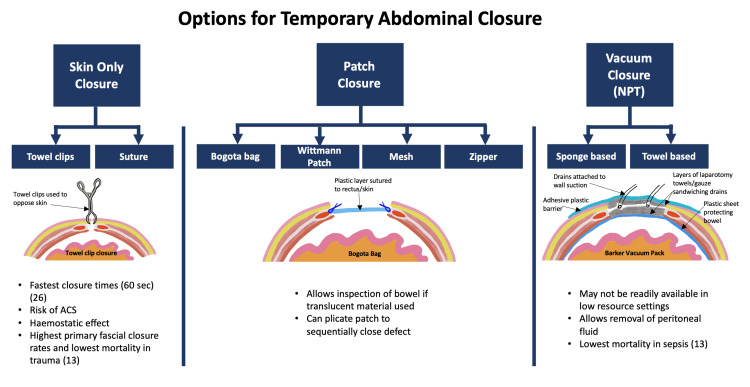
Infographic showing options for temporary abdominal closure

The techniques for TAC can be broadly grouped into three categories displayed in Figure 4. The categories are skin-only closure, patch closure, and vacuum closure (NPT).

Skin-only closure techniques involve closing the skin and leaving the fascia open. This is most commonly achieved using towel clips or suture material to close the skin. Many sources, including consensus guidelines, reviews, and textbooks, suggest that skin closure techniques should be abandoned [6-11]. Arguments against skin-only closure include an inability to control abdominal effluents, interference with radiographic imaging (as observed in the index case), high incidence of ACS, failure to prevent loss of domain, high incidence of evisceration, and the high incidence of skin complications. Reviewing the references for the assertions that skin-only techniques should be abandoned reveals a limited exploration of the available data. We are of the opinion that skin-only closure deserves a second look. Furthermore, much of the data on skin only closure was collected before the era of damage control resuscitation. The use of more conservative resuscitation methods may alter complications associated with TAC, especially the incidence of ACS [24].

Table 1 summarises the data from published series on skin-only closure for OA. Overall skin closure for TAC is associated with a high fascial closure rate of 82% with a moderate risk of enterocutaneous fistula (6%) and ACS (18.5%). Caution is necessary when interpreting these data as they are retrospective, highly heterogeneous and many of the studies did not include important outcomes. Nevertheless, good results are possible with skin-only closure, as was demonstrated in the index case. The incidence of skin complications is largely unreported. It seems intuitive that repeated application of clips to the skin would increase the risk of infection and skin complications, making skin-only closure with towel clips unsuitable for patients in whom multiple return trips to the operating theatre are anticipated. However, further research is needed to clarify this issue.

**Table 1:** The outcomes of 723 cases of open abdomen managed with skin only closure (for TAC) ^†^Reported skin closure and Bogota bag closure together. - Data not reported in the study. OA: open abdomen, ECF: enterocutaneous fistula, ACS: abdominal compartment syndrome, TAC: temporary abdominal closure.

Author	Year	Indication for OA	Number of patients	Method of closure	Fascial closure rate (%)	Average time to fascial closure (days)	Incidence of ACS (%)	Incidence ECF (%)
Burch et al. [25]	1991	Trauma	189	Suture/towel clips	-	-	-	4
Smith et al. [26]	1992	Trauma	6	Towel clips	100	-	-	-
Offner et al. [27]	2001	Trauma	25	Towel clips	-	-	24	-
Raeburn et al. [28]	2001	Trauma	47	Suture/ towel clips	-	-	36	-
Tremblay et al. [29]	2001	Trauma and non-trauma	93	Suture/towel clips	66	-	14	4
Patel et al. [30]	2011	Trauma and non-trauma	28	-	97	3	-	-
Coccolini et al. [31]^†^	2017	Trauma and non-trauma	117	-	71	5		7.4
Hu et al. [24]	2018	Trauma	138	Suture	96.4	3.35	-	2.9
Brigode et al. [32]	2019	Trauma	55	-	62	-	-	-
Kruger et al. [33]	2021	Trauma and non-trauma	25	Suture	84	-	0	12
Average					82.3	3.66	18.5	6

Skin-only closure is quick, readily available, and inexpensive [9]. It can often be completed in as little as 60 seconds which is beneficial in the hemodynamically unstable patient, allowing the rapid termination of a laparotomy as was seen in the index case [25]. Expeditious closure of the abdomen also reduces operating time, which has cost-saving implications that may be particularly relevant in low-resource settings. Closing the skin reduces heat and fluid losses [34]. The anatomical attachments of the skin and subcutaneous tissue to the underlying muscles may help prevent loss of domain when skin only closure is employed [32].

In a retrospective analysis comparing 239 patients undergoing DCS for trauma, patients who had TAC with skin-only closure were compared to those who had TAC with Bogota bag, ABThera™ VAC system (KCI USA, Inc., San Antonio, TX), and Barker’s vacuum-packing. Patients with skin-only closure had higher primary fascial closure rates and lower mortality than those with TAC with the other techniques [24].

Examining ACS specifically, skin-only closure is associated with a risk of ACS due to the limited ability of the approximated skin to stretch in compliance with worsening visceral oedema [24]. However, regardless of the method of TAC, the risk of ACS is ever-present for most patients requiring an OA. Head-to-head comparisons of primary fascial closure, Bogota bag, and skin-only closure have revealed an equivalent risk of ACS in patients undergoing DCS [28]. Furthermore, the increased intraabdominal pressure that may be observed with skin-only closure is likely to facilitate hemostasis in patients who have ongoing bleeding after DCS [35]. This may explain why preliminary data from the International Register of Open Abdomen revealed that skin-only closure and Bogota bags are associated with higher primary fascial closure rates and lower mortality for trauma patients when compared to NPT [31]. As the register accrues more data, it is hoped that it can further elucidate the role of skin-only closure by reporting patient results separate from those who underwent Bogota bag TAC. To date, there is no level one evidence exploring the role of skin only closure. Available data need to be interpreted with caution.

Patch TAC techniques include the Bogota bag, mesh closure, Wittmann patch, and zipper closure. Patch techniques provide a barrier between the peritoneum and the external environment, which reduces fluid and heat loss. Compared to vacuum closure techniques, they are not effective at removing peritoneal fluid, which is particularly important for patients undergoing TAC for sepsis. Further, while patch closure allows expansion of intrabdominal content beyond the normal anatomical confines, the expansion is limited to a fixed volume based on the size of the patch. Thus, it is still possible to develop ACS [36].

Bogota bag use was first documented by Mattox, who observed Colombian surgeons achieving TAC by suturing empty intravenous fluid bags to the edge of laparotomy wounds when fascial closure was not possible [37]. Surgical drapes and silastic cloth may be employed as an alternative to intravenous fluid bags [15]. The process of suturing the chosen material to fascia may predispose it to injury [7], and surgeons may choose to affix the patch to the skin [38]. The use of the Bogota bag spread rapidly because of its low cost, ease of use, and the widespread availability of the necessary materials [22]. When translucent patch material is used, it has the added benefit of allowing inspection of intrabdominal contents without opening the TAC. Bogota bags may not be particularly effective at preventing loss of domain, with a wide range of primary fascial closure rates reported in the literature from 12% to 82% [9].

As early as the 1980s TAC using mesh was described to facilitate abdominal re-exploration [15]. Mesh can be sutured to the fascia facilitating a tension-free TAC in a similar manner to the Bogota bag. The mesh can then be removed at the time of re-operation. In cases where fascial closure cannot be achieved at the first re-visit, the mesh can be trimmed in the middle, and fascial edges bought closer together by re-apposing the edges of the mesh. Various types of mesh have been employed. The use of polypropylene mesh has largely been abandoned due to the high risk of enterocutaneous fistulae occurring in up to 34% of cases [39]. Polytetrafluoroethylene mesh has the advantage of being less adherent to the bowel compared to polypropylene. However, the micropores in the mesh allow bacteria to colonise and evade host cells leaving the laparostomy at high risk for infection [9]. Absorbable meshes, including polyglactin and polyglycolic acid, have been used to good effect, with lower enterocutaneous fistula rates than polypropylene (5-11%) [9]. When primary fascial closure is impossible, the use of absorbable mesh encourages the formation of a granulation tissue bed, which facilitates skin grafting [9]. Non-absorbable mesh TAC is associated with a primary fascial closure rate of 33-89% [36].

Wittman et al. described using Velcro-like material to achieve TAC for patients with intraabdominal sepsis in 1990 [40]. Two sheets of material are sutured to the abdominal fascia. One sheet has small loops and the other small hooks which engage with each other and keep the abdomen closed. Abdominal re-entry is facilitated by separating the sheets. The sheets can be sequentially advanced over each other and the excess trimmed to facilitate fascial closure. At the time of definitive fascial closure, the sheets are removed. The Wittman patch achieves primary fascial closure in 65.7-100% of cases with an incidence of enterocutaneous fistula ranging from 0% to 42% [31,36]. While the Wittman patch is effective, the cost is prohibitive ($1,440.00 USD per patient) [41].

Zipper closure uses a sterile zipper that is sutured to the edges of the fascia and the zip closed to contain intrabdominal contents. Leguit first described its use in 1982 [42]. Stone et al. further popularised the technique after describing the use of zippers to facilitate repeated abdominal entry in treating patients with pancreatic abscess [43]. It is easy to apply and facilitates rapid re-entry into the abdomen. The technique fell out of vogue after the 1980s. However, two recent papers report primary fascial closure rates of 50-100% [44,45].

NPT TAC utilises an airtight seal that is attached to a vacuum pump. The negative pressure exerts inward traction on the abdominal wall resisting loss of domain and increasing the likelihood of primary fascial closure [15]. There is an improvement of local blood supply which increases the partial pressure of oxygen in the wound and decreases the proliferation of bacteria. The cytokine milieu of wounds changes in response to negative pressure. Increased levels of vascular endothelial growth factor and interleukin-8 are observed, which promotes angiogenesis and chemoattracts neutrophils [46].

NPT also effectively removes third-space fluid from the abdomen. This is particularly useful in patients who have an OA for sepsis. The removal of infected fluid and inflammatory mediators has a positive impact on patient outcomes. Septic patients treated with NPT TAC have improved mortality compared to those with TAC by other methods [31]. The benefits of NPT's ability to clear third space fluid are further highlighted by the impressive results obtained when it is coupled with peritoneal resuscitation, which is dependent on the negative pressure to clear the resuscitation fluids. A randomised controlled trial was done on patients who had negative pressure TAC after DCS. It compared patients who had standard resuscitation versus those who had peritoneal resuscitation with glucose-based peritoneal dialysis solution. Patients who had peritoneal resuscitation had higher rates of primary fascial closure (83% vs 66%, p ≤ 0.05), lower thirty-day mortality (13% vs 28%), and reduced intrabdominal complications (8% vs 18%) [47].

Consensus guidelines are unanimous in their recommendation that NPT is the TAC technique of choice, advising against other techniques because of low facial closure rates and high incidence of enterocutaneous fistulae [5,7,12]. NPT can be made using laparotomy towels or using commercially available sponge-based systems.

The vacuum pack of Barker and coworkers was initially described in 1995 [48]. Its application is performed as follows. A polythene sheet is fenestrated and placed between the anterior parietal peritoneum and the abdominal viscera. Above the sheet, moist laparotomy towels are placed. Two closed suction drains are placed above the towels. More laparotomy towels are placed over the drains, and the entire wound is then covered with an adhesive dressing to form an airtight seal. The drains are attached to suction, creating a negative pressure system set at 100-150 mmHg (Figure 4). This system has the advantage of employing consumables that are widely available and are low cost ($50.00 USD per patient) [49]. Even with the low cost of consumables, this form of TAC may still be unavailable in low-resource settings where access to suction devices is not universal. Barker et al. [49] reported their experience using this form of NPT in 242 trauma and general surgery patients. Primary fascial closure was achieved in 68.1% of patients with enterocutaneous fistulae and ACS developing in 5% and 1.2%, respectively.

Commercial negative pressure systems using polyurethane sponges include the VAC® and ABThera™ systems. Similar to the Barker vacuum pack, the underlying viscera is protected by a fenestrated polythene sheet. Foam is placed between the sides of the laparotomy wound and then covered with an adhesive dressing to form a seal. ABThera™ uses a radial sponge design that extends down to the paracolic gutters. Comparing commercial systems to the Barker vacuum pack reveals similar fascial closure rates, a similar incidence of enterocutaneous fistulas but lower mortality in those treated with commercial systems [50,51]. The reason behind this difference in mortality is yet to be elucidated. The cost of commercial systems can be prohibitive with a large sponge and canister VAC® dressing costing $72.14 USD, the ABThera™ sponge costing $350 USD, and the rental cost of the portable suction machine costing $67.50 USD per day [41,52].

In summary, the data on TAC are heterogeneous and must be carefully interpreted before deciding on which technique to use for individual patients. We disagree with the trend in the literature to dismiss skin-only closure techniques as a treatment option. The data cannot equivocally demonstrate a single superior technique. The varying indications for TAC have different pathophysiology, and this must be taken into consideration. The surgeon should not default to using the most advanced technique available but should consider the individual patient's circumstances. NPT may be superior for patients with sepsis, while the skin-only closure technique may be more beneficial in trauma patients [31,51].

We look forward to the publication of further data from the International Register of Open Abdomen and encourage surgeons to include their patients in the register. With more data, it is hoped that we can achieve a greater understanding of the best ways to manage the challenging clinical condition that is the OA.

## Conclusions

DCS is facilitated by TAC to maintain an OA until patients are physiologically ready for definitive repair. There are numerous techniques available for TAC. This case report describes the use of towel clips to achieve TAC after DCS for multiple gunshot wounds. While many sources suggest that skin-only closure should be abandoned, a review of the literature reveals that it can result in good outcomes, similar to the index case. Surgeons should carefully consider the literature on TAC and the pathophysiology of individual patients before selecting a TAC method.
